# Insights into Divergent Leishmaniasis Pathogenesis: A Quantitative Flagellar Proteomic Comparison of *L. mexicana*, *L. amazonensis*, and *L. infantum*

**DOI:** 10.3390/microorganisms14071411

**Published:** 2026-06-26

**Authors:** Diya Lalu Patel, Seok-Young Kim, Olga Uchakina, Niani Angelle Clermont, Jean Byung Hyun, Aleem Damji Patni, Manas Paresh Patel, Shan Khan, Dhruv K. Rana, Chan Hyun Na, Sung-Jae Cha

**Affiliations:** 1 Department of Biomedical Sciences, Mercer University School of Medicine, 1501 Mercer University Drive, Macon, GA 31207, USA; diya.lalu.patel@live.mercer.edu (D.L.P.); uchakina_o@mercer.edu (O.U.); niani.angelle.clermont@live.mercer.edu (N.A.C.); jean.byung.hyun@live.mercer.edu (J.B.H.); aleem.damji.patni@live.mercer.edu (A.D.P.); manas.paresh.patel@live.mercer.edu (M.P.P.); shan.khan@live.mercer.edu (S.K.); dhruv.k.rana@live.mercer.edu (D.K.R.); 2 Department of Neurology, Institute for Cell Engineering, School of Medicine, Johns Hopkins University, Baltimore, MD 21205, USA; skim501@jhmi.edu

**Keywords:** metacyclic promastigotes, cutaneous leishmaniasis, mucocutaneous leishmaniasis, visceral leishmaniasis, multigene family, flagellar-surface virulence factors

## Abstract

Leishmaniasis, a neglected tropical disease endemic to 98 countries, affects more than 300 million people worldwide. The disease is transmitted through the bite of infected female phlebotomine sand flies harboring *Leishmania* parasites. Although infection initiates at the cutaneous inoculation site, *Leishmania* species exhibit distinct tissue tropisms, resulting in three primary clinical manifestations: cutaneous (CL), mucocutaneous (MCL), and visceral leishmaniasis (VL). The initial stage of infection involves the engulfment of metacyclic promastigotes (MPs) by host phagocytes, a process mediated by early interactions between the MP flagellum and the host cell surface. This study investigates how species-specific MP-flagellar proteomic profiles dictate unique interactions with host cell populations, thereby driving divergent pathogenic outcomes. To address this, we conducted a comparative quantitative proteomic analysis of flagella from *Leishmania* species representing each clinical form. Our analysis revealed distinct flagellar proteomic signatures that differentiate the VL-causing species, *L. infantum*, from others. Notably, we identified five virulence factor families that were differentially expressed: amastins, cysteine peptidases, heat shock proteins, promastigote surface antigens, and leishmanolysins. These findings link flagellar surface composition to species-specific pathogenicity, providing molecular insights into early infection dynamics and identifying potential antigenic targets for developing species-specific vaccines and therapeutics.

## 1. Introduction

Leishmaniasis is caused by protozoa of the genus *Leishmania*, which belongs to the family Trypanosomatidae and comprises approximately 22 species that infect humans across two subgenera: *Leishmania* and *Viannia* [1,2]. Leishmaniasis accounts for an estimated 700,000 to 1 million new cases annually, resulting in 30,000 deaths each year [1,3,4]. Global warming and human activities increase the risk of transmission, and the USA has been classified as endemic for leishmaniasis since 2015 due to autochthonous cases [5].

Cutaneous leishmaniasis (CL) is the most common form, with an estimated 600,000 to 1 million new cases annually, causing ulcer-like skin lesions that leave lifelong scars, severe disability, and stigma [6,7,8]. Mucocutaneous leishmaniasis (MCL) induces granulomatous and destructive mucosal lesions in the oral, nasal, and pharyngeal cavities. It primarily occurs in “New World” countries, with approximately 1700 new cases reported annually [9]. Of the three forms of leishmaniasis, Visceral leishmaniasis (VL) is the most devastating, accounting for roughly 50,000–90,000 cases annually [10]. VL causes fever, hepatosplenomegaly, and pancytopenia, which can lead to 30,000 deaths annually [4]. Treating the disease requires a competent host immune system, as medicines alone will not eliminate the parasites from the body, increasing the risk of relapse in immunosuppressed cases [8]. No preventive vaccine is available for humans, and treatment options are limited by cost, toxicity, and availability [5]. Developing new drugs and vaccines for controlling leishmaniasis is a high priority.

*L. mexicana* is primarily endemic to Central America, Mexico, and northern South America, where it typically causes localized CL. In contrast, *L. amazonensis*, prevalent in the Amazon basin, presents a broader clinical spectrum, including diffuse cutaneous leishmaniasis. While MCL is typically characteristic of the subgenus *Viannia*, it is worth noting that *L. amazonensis* is more frequently associated with mucosal involvement than other members of the *L. mexicana* complex in certain instances. *L. infantum* possesses a broader geographic range—spanning the Mediterranean, Middle East, Asia, and Latin America—and is the primary etiological agent of severe VL (kala-azar), which is characterized by systemic symptoms and high mortality rates if untreated. Although these three species co-exist in the New World, they are distinguished by their unique clinical manifestations [11,12]. Although the disease spectrum is species-specific, all *Leishmania* infections begin with a metacyclic promastigote (MP) invasion of phagocytic mononuclear cells at the bite site.

The delivery of flagellated motile MPs from the proboscis of the infected sand fly to the bite site initiates infection in the vertebrate host by *Leishmania* parasites [13]. MPs enter phagocytic mononuclear host cells at the bite site, including neutrophils, monocytes, mast cells, natural killer cells, dendritic cells, and skin macrophages [7,14]. Inside the phagolysosome, the flagella of MPs disappear, and parasites transform into amastigotes, which then multiply and develop within the reticuloendothelial system [5,14]. Therefore, MP infection of phagocytic host cells is the essential first step for disease development and is a key target for immunization. Notably, the route of parasite distribution in the body varies by species, which explains the species-specific forms of the disease (CL, MCL, and VL). Different *Leishmania* species exhibit distinct surface molecules, resulting in unique interactions between these parasite surface molecules and host cell receptors for each parasite-host pair [15,16]. The flagella stimulate host cell phagocytosis. A video microscopy study shows that 65% of MPs are engulfed by the host cell through flagella-host cell membrane interactions, 29% are internalized via flagella base-host cell interactions, and only 6% are internalized through body-host cell interactions [14]. MP entry into phagocytic host cells is believed to be mediated by receptor-mediated phagocytosis [16]. Furthermore, MPs of a particular species can interact with different molecules on various host cells, triggering other downstream host cell responses [16]. Therefore, characterizing the initial ligand-receptor interactions for each parasite species can provide mechanistic insights into disease pathways and help identify candidate MP antigens for vaccines.

We propose that *Leishmania* infection initiates through the entry into phagocytic cells—a process mediated by MP flagellar ligands—thereby triggering species-specific disease pathways. This is supported by our peptide-display library screening data, which shows that distinct MP-binding peptides were identified against two different *Leishmania* species, and these peptides inhibit MP-host cell invasion in a species-specific way [17]. We collected flagella from MPs of *L. amazonensis*, *L. mexicana*, and *L. infantum* for quantitative proteomic analysis. Notably, the flagella of VL-causing *L. infantum* exhibit distinct protein profiles compared to those of the other two species. We analyzed the flagellar surface proteins, focusing on five differentially expressed virulence factors: amastin, cysteine peptidase (CP), heat-shock protein (HSP), promastigote surface antigen (PSA), and leishmanolysin (also known as GP63) [18,19]. These candidates comprise multigene families [18,20,21,22,23,24,25,26,27], many of which have shown promise as vaccine antigens. However, our quantitative analysis reveals that the majority of these highly expressed, species-specific proteins remain unexplored as potential vaccine or drug targets.

## 2. Materials and Methods

### 2.1. Parasite MP Culture

*L. amazonensis* (MPRO/BR/1972/M1841-LV-79), *L. mexicana* (MNYC/BZ/62/M379), and *L. infantum* (MHOM/MA/67/ITMAP-263) MPs were prepared from stationary axenic cultures in Schneider’s Insect Medium (Sigma-Aldrich, St. Louis, MO, USA) with L-glutamine, supplemented with 10% heat-inactivated fetal bovine serum (FBS, Gibco, Grand Island, NY, USA), 2.5 μg/mL hemin (for *L. infantum* only), and 1% antibiotics (100 U/mL penicillin, 100 μg/mL streptomycin) at 26 °C.

### 2.2. CFSE Stain

Parasites were visualized with CFSE (eBioscience, San Diego, CA, USA), as described previously [17]. Briefly, *Leishmania* parasites were harvested and washed twice with phosphate-buffered saline (PBS). Then, 5 × 10^8^ parasites/mL were incubated with 5 μM CFSE in PBS for 15 min in a dark 26 °C chamber. After incubation, the parasites were harvested by centrifugation for 5 min at 800× *g* and washed twice with ice-cold PBS.

### 2.3. Deflagellation of MPs

5 × 10^8^ *Leishmania* MPs from a stationary culture were collected by centrifugation at 800× *g* for 15 min at 4 °C, then washed with PBS. MPs were resuspended in 5 mL of deflagellation buffer (10 mM PIPES, 10 mM NaCl, 75 mM CaCl_2_, 1 mM MgCl_2_, 0.32 M sucrose, pH 7.2), with protease inhibitor cocktail (Thermo Scientific, Waltham, MA, USA). All steps were performed on ice to prevent proteolytic degradation. Cells were deflagellated using mechanical shear, and the flagella were separated from the cell bodies following a protocol with minor modifications [28]. Briefly, the cell suspension was drawn 100 times through the tip of a 200 µL protein gel loading pipette attached to a 10 mL syringe until the flagella were sheared from the cell bodies. The mixture was layered onto a 33% sucrose solution and centrifuged at 800× *g* for 15 min at 4 °C without a break. The cell bodies pelleted at the bottom, and the top layer containing the flagella was transferred to another tube with a 33% sucrose layer. After an additional sucrose sedimentation at 800× *g* for 15 min at 4 °C, the top layer was collected and transferred to a new centrifuge tube. The flagella were then pelleted by centrifugation at 21,000× *g* for 30 min at 4 °C. Flagellar purity was confirmed by microscopy. The isolation procedure was independently replicated five times for each species.

### 2.4. Sample Preparation for Mass Spectrometry

For protein extraction, 200 µg of washed flagella pellet was reconstituted in a lysis buffer (10 M urea and 100 mM triethylammonium bicarbonate [TEABC] Sigma-Aldrich, St. Louis, MO, USA) to reach final concentrations of 5 M urea and 50 mM TEABC. The samples were then reduced and alkylated using 10 mM TCEP (tris(2-carboxyethyl) phosphine) and 40 mM CAA (chloroacetamide) for 1 h at room temperature. Sequential proteolytic digestion was performed, starting with LysC (Lysyl endopeptidase, mass spectrometry grade, Fujifilm Wako Pure Chemical Industries Co., Osaka, Japan) at 10 ng/µL for 3 h at 37 °C. After diluting the urea concentration to 2 M with 50 mM TEABC, trypsin (Promega, Madison, WI, USA, 10 ng/µL) was added for overnight incubation at 37 °C. The resulting peptides were acidified to 1% trifluoroacetic acid (TFA), desalted using C18 StageTips, and vacuum-dried using a SpeedVac (Thermo Fisher Scientific, Waltham, MA, USA). For multiplexed quantification, peptides were labeled with TMTpro 18-plex reagents (Thermo Fisher Scientific, Waltham, MA, USA) according to the manufacturer’s instructions by incubating peptides with labeling reagents for 1 h at room temperature. The reaction was quenched with 1/10 volume of 1 M Tris-HCl (pH 8.5) for 15 min. Labeled samples were pooled and vacuum-dried. Offline high-pH reversed-phase fractionation was conducted by reconstituting peptides in 10 mM TEABC (pH 8.5) and separating them on an Agilent 300Extend-C18 column (5 µm, 4.6 mm × 250 mm; Agilent Technologies, Santa Clara, CA, USA) using an Agilent 1260 LC system (Agilent Technologies, Santa Clara, CA, USA). Peptides were eluted with a gradient of solvent B (10 mM TEABC in 90% acetonitrile, pH 8.5) at a flow rate of 0.3 mL/min. Peptides were collected into 96 fractions over 150 min and concatenated into 24 superfractions. The fractions were then vacuum-dried and stored at −80 °C until LC-MS/MS analysis.

### 2.5. Mass Spectrometry Analysis

Liquid chromatography–tandem mass spectrometry (LC-MS/MS) was performed as previously described [29] with minor modifications. Fractionated samples were analyzed on an Orbitrap Fusion Lumos Tribrid Mass Spectrometer (Thermo Fisher Scientific, San Jose, CA, USA) coupled to an UltiMate 3000 RSLCnano liquid chromatography system (Thermo Fisher Scientific). Reconstituted samples (0.1% formic acid [FA]) were loaded onto an Acclaim PepMap 100 C18 trap column (5-µm, 100 µm × 20 mm; Thermo Scientific, Waltham, MA, USA) at 8 µL/min. Subsequent peptide separation was achieved on an EASY-Spray PepMap RSLC C18 analytical column (2-µm, 75 µm × 500 mm; Thermo Fisher Scientific, Waltham, MA, USA) at 0.3 µL/min using a 120-min linear gradient of 0.1% FA in water and 0.1% FA in 95% acetonitrile. Data-dependent acquisition (DDA) was used with a top-speed method, acquiring MS1 precursor scans over an *m*/*z* range of 300–1800 at a resolution of 120,000 (at *m*/*z* 200). The automatic gain control (AGC) target was set to 1 × 10^6^ with a maximum injection time of 50 ms. For MS2 analysis, the most intense precursor ions with charge states of 2–5 were selected within a 3-s cycle for fragmentation via higher-energy collisional dissociation (HCD) at a normalized collision energy (NCE) of 35%. Fragment ions were detected at a resolution of 50,000 (at *m*/*z* 200). The MS/MS AGC target was 5 × 10^4^ with the ion filling time of 100 ms. Precursors were isolated using a 1.6 *m*/*z* window with a 0.4 *m*/*z* offset. Dynamic exclusion was enabled for 30 s, and singly charged ions were excluded. Mass spectra were internally calibrated using the ambient air lock mass (*m*/*z* 445.1200025).

### 2.6. Proteomics Data Analysis

Proteomic data were processed using Proteome Discoverer (v3.2.0.450, Thermo Fisher Scientific) following a previously established protocol [29]. The tandem mass spectrometry data were analyzed using the SEQUEST algorithm against a *Leishmania* database from UniProt (comprising Swiss-Prot and TrEMBL entries for *L. mexicana*, *L. amazonensis*, and *L. infantum*; downloaded in May 2026), supplemented with common contaminant proteins. Search parameters specified trypsin as the protease with up to two missed cleavages, a precursor mass tolerance of 10 ppm, and a fragment mass tolerance of 0.02 Da. Carbamidomethylation of cysteine residues (+57.02146 Da) and TMTpro labeling (+304.207146 Da) on lysine residues and peptide N termini were defined as fixed modifications, whereas oxidation of methionine (+15.99492 Da) was included as a variable modification. A minimum peptide length of six amino acids was required. A 1% false discovery rate (FDR) threshold was applied at both the peptide and protein levels using Percolator for peptide-spectrum match (PSM) validation and the Protein FDR validator node for protein-level filtering. Protein quantification was conducted using the most confident centroid integration mode and the reporter ion tolerance of 20 ppm. Quantification was performed at the MS2 level using HCD fragmentation. Only unique peptides were considered for quantification, with protein groups utilized to assess peptide uniqueness. Reporter ion abundance was determined based on signal intensity, applying an average reporter ion signal-to-noise (S/N) threshold of 10% and a co-isolation interference threshold of 50%. Protein grouping was performed based on a strict parsimony principle. Specifically, proteins sharing identical or subset peptide evidence were clustered, whereas those lacking unique peptide support were excluded. Proteome Discoverer finalized these protein groups through iterative spectral analysis, prioritizing peptide-spectrum matches (PSMs) with the highest number of unambiguous and unique peptides [29].

### 2.7. Statistical and Bioinformatics Analysis

The statistical analysis of the MS data was performed using Perseus 1.6.7.0 [30]. Protein abundance data from the TMT experiment were log2-transformed and sequentially normalized through sample-wise and protein-wise median centering. First, sample-wise median centering was performed by subtracting the column median from the transformed values. Subsequently, protein-wise median centering was applied by subtracting the row median from the corresponding abundance data. Statistical significance between comparison groups was evaluated using Student’s two-sample *t*-test. Proteins with q-values below 0.05 were considered differentially expressed. The *q*-values for the volcano plot were calculated using significance analysis of microarrays (SAM) with permutation-based FDR estimation, with a S0 value of 0.1 [31]. Principal component analysis (PCA) and heatmap visualizations were performed using the MetaboAnalyst tool (ver. 6.0) [32]. PCA was performed using the sample-wise median-centered abundance values. In contrast, heatmap analysis utilized data subjected to both sample-wise and protein-wise median centering, from which the top 300 differentially expressed proteins (DEPs) were selected based on one-way ANOVA with false discovery rate (FDR) correction. Protein–protein interaction (PPI) network and pathway analyses were conducted with STRING (version 12.0) [33]. Flagellar surface proteins were identified by the presence of a signal peptide, transmembrane domains, or a GPI anchor using bioinformatics tools (https://services.healthtech.dtu.dk/services/SignalP-5.0/; https://tmdas.bioinfo.se/; https://services.healthtech.dtu.dk/services/NetGPI-1.1/, accessed on 16 February 2026). Using multiple sequence alignment (https://www.genome.jp/tools-bin/clustalw, accessed on 16 February 2026) and phylogenetic analysis with the ETE3 pipeline (https://www.genome.jp/tools-bin/ete, accessed on 16 February 2026), amastins were classified into four groups, as previously described [25].

## 3. Results

### 3.1. Flagella Isolation and Proteome Analysis

**Figure 1:**
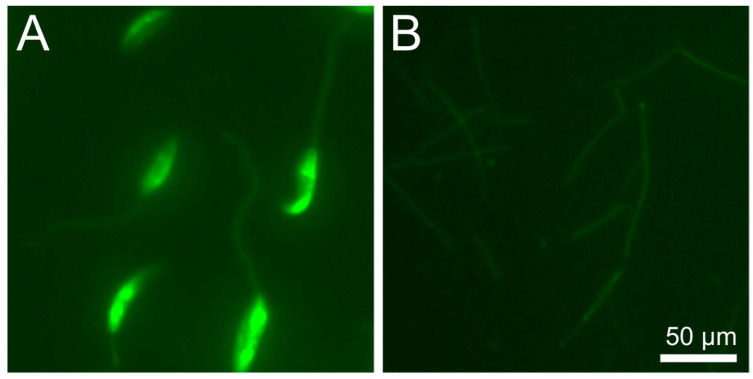
Flagella isolation. (**A**) Axenically cultured *L. infantum* MPs were stained with CFSE. (**B**) MP flagella were isolated using mechanical shearing and sucrose density gradient centrifugations.

To identify proteomic profiles, flagella of MPs were isolated from a stationary axenic culture of each species as previously described [28]. Parasites and isolated flagella were visualized with carboxyfluorescein diacetate succinimidyl ester (CFSE) staining (Figure 1). Mass spectrometry analysis of the differentially labeled flagella detected *Leishmania*-specific peptides and their abundance (Table S1). The associated proteins were identified using the *Leishmania* UniProt database, and the relative abundances of the two species were summarized in Table S2.

### 3.2. Proteomic Profiling Reveals Species-Specific Signatures in Leishmania flagella

**Figure 2:**
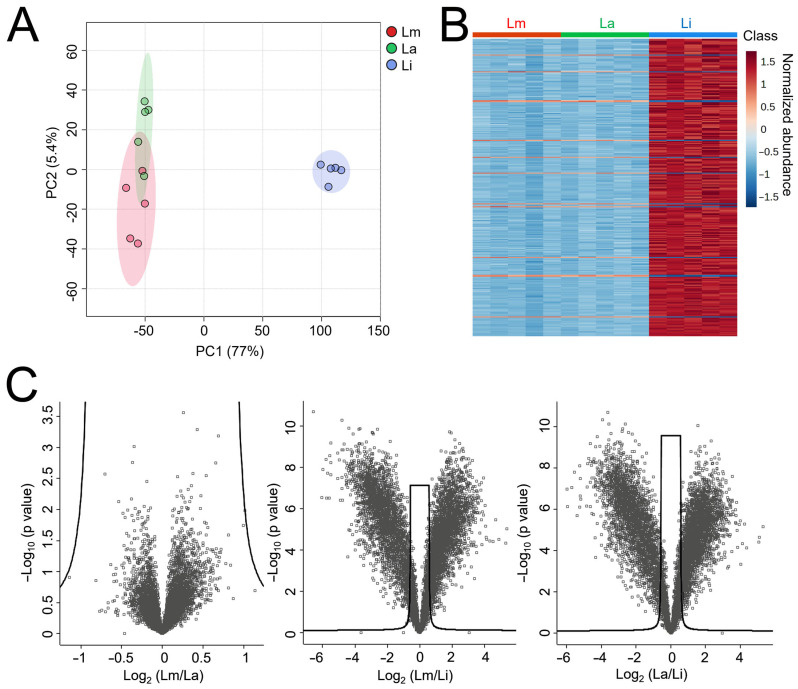
Proteomic profiling of three *Leishmania* species (*L. mexicana*, *L. amazonensis*, and *L. infantum*) using TMT-based LC-MS/MS analysis. (**A**) The PCA plot shows proteomic differences among the three species. PC1 and PC2 are the main components that capture the most variation in the data. The red, green, and blue ellipses represent the 95% confidence regions for the corresponding group clusters. La, *L. amazonensis*; Lm, *L. mexicana*; Li, *L. infantum.* (**B**) The heatmap displays hierarchical clustering of protein expression, highlighting distinct proteomic patterns among the species. (**C**) The volcano plots illustrate proteins that are differentially expressed between each pairwise comparison. Proteins outside the curved lines have a *q*-value < 0.05, indicating statistically significant differences.

To assess the overall expression profile similarities and differences among three *Leishmania* species [*L. mexicana*, *L. amazonensis*, and *L. infantum*], the flagella fractions from each species were analyzed using tandem mass tag (TMT) proteomic analysis followed by principal component analysis (PCA) [34]. Notably, the proteome of *L. infantum* showed a distinct pattern compared to those of *L. mexicana* and *L. amazonensis*, while *L. mexicana* and *L. amazonensis* had highly similar profiles (Figure 2A). This indicates that *L. mexicana* and *L. amazonensis* share closer genetic or transcriptomic traits, whereas *L. infantum* is markedly different. These observations were further supported by hierarchical clustering of protein expression profiles (Figure 2B). The heatmap revealed that *L. mexicana* and *L. amazonensis* clustered together, while *L. infantum* formed a separate branch, consistent with the PCA results. Volcano plot analysis also confirmed these findings. No significantly differentially expressed proteins (*q* < 0.05) were identified between *L. mexicana* and *L. amazonensis* (Figure 2C). In contrast, many differentially expressed proteins were detected between *L. infantum* and either *L. mexicana* or *L. amazonensis*.

### 3.3. Protein–Protein Interaction Network Analysis Between L. amazonensis and L. infantum

**Figure 3:**
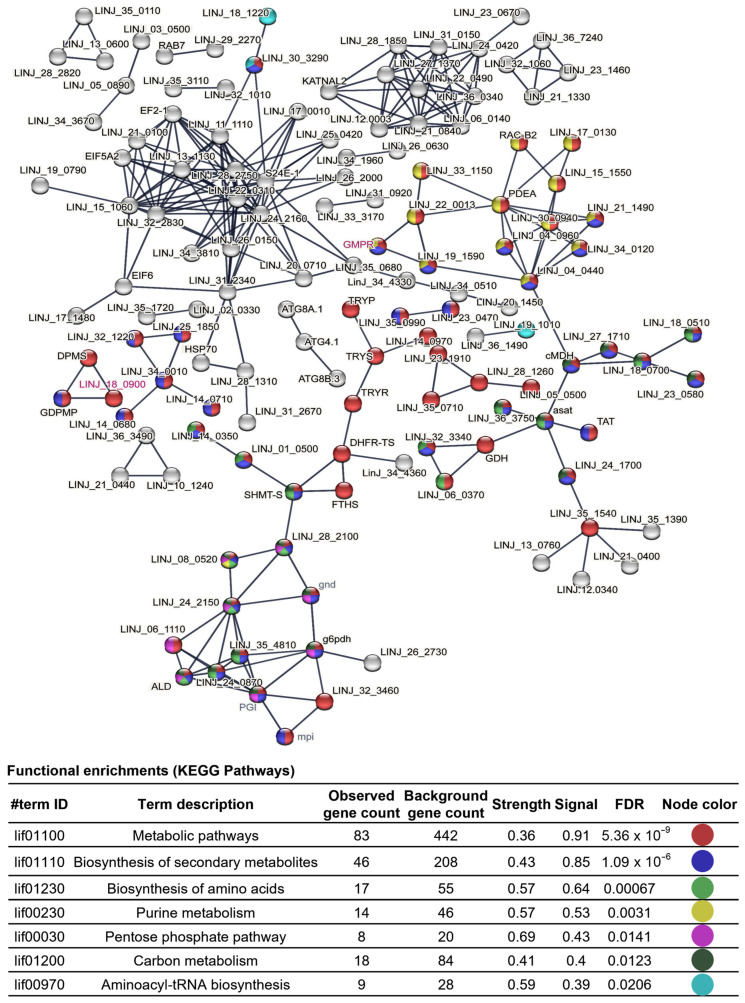
STRING protein-protein interaction (PPI) network and functional enrichment analysis of differentially expressed proteins (DEPs) between *L. amazonensis* and *L. infantum*. The network exhibits an average node degree of 0.777, an average local clustering coefficient of 0.154, and a PPI enrichment *p*-value of 1.34 × 10^−12^. A highest-confidence interaction score threshold of ≥0.9 was applied. The inset table summarizes the enriched KEGG pathways. Node coloring denotes pathway associations: red, metabolic pathways; blue, biosynthesis of secondary metabolites; green, biosynthesis of amino acids; yellow, purine metabolism; pink, pentose phosphate pathway; dark green, carbon metabolism; sky blue, aminoacyl-tRNA biosynthesis; and grey, proteins unassigned to enriched pathways. For pathway-associated nodes, text colors indicate summarized gene-level abundance changes: pink, higher in *L. amazonensis*; black, higher in *L. infantum*; gray, discordant protein-level changes. # indicates the KEGG pathway identification (ID).

Since no significant proteome differences were identified between *L. mexicana* and *L. amazonensis* flagella, further analyses focused on the differences between *L. infantum* and *L. amazonensis*. To investigate protein–protein interactions and identify potential hub proteins among the differentially expressed flagella proteins in *L. amazonensis* and *L. infantum*, we conducted a STRING-based PPI network analysis [33]. After removing disconnected nodes, the network included 664 proteins (nodes) and 258 interactions (edges), based on the top 2000 differentially expressed proteins (Figure 3). Due to the limited annotation of *Leishmania* proteins in the STRING database, 675 proteins were successfully mapped and included in the analysis. Most interacting proteins within clusters were more abundant in the *L. infantum* flagella proteome compared to *L. amazonensis*, suggesting unique features of *L. infantum* flagella that could serve as potential biomarkers for species identification. KEGG pathway enrichment revealed seven major pathways: metabolic pathways, biosynthesis of secondary metabolites, biosynthesis of amino acids, purine metabolism, pentose phosphate pathway, carbon metabolism, and aminoacyl-tRNA biosynthesis, indicating functional processes that may be linked to the observed proteomic differences [35].

### 3.4. Differentially Expressed Flagella-Surface Virulence Factors

**Figure 4:**
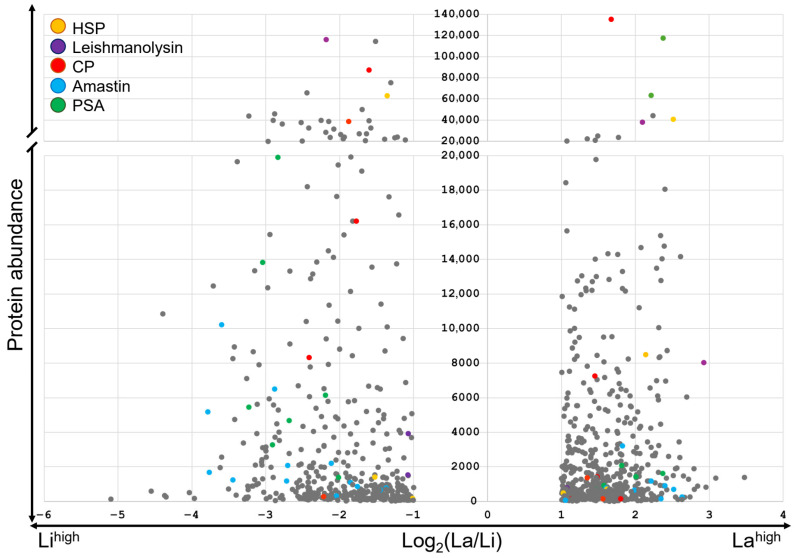
Differentially expressed MP flagella-surface virulent factors between *L. infantum* and *L. amazonensis*. The variation in flagella surface protein levels is plotted as the relative ratio (Log_2_La/Li) on the *x*-axis against protein abundance on the *y*-axis. Specific flagella-surface virulence factors are color-coded as indicated in the legend. Detailed protein abundance and log_2_ ratio values are provided in Table S3.

To understand how interactions between flagellar surface proteins and host cells drive species-specific manifestations of leishmaniasis, we focused on identifying proteins differentially expressed on the flagellar membrane of *L. infantum* and *L. amazonensis*. A total of 1015 flagellar surface proteins were identified using bioinformatics tools, and their abundance and differential expression, calculated as log_2_(La/Li), are summarized in Table S3. Of these, 559 proteins were enriched in *L. amazonensis* MP flagella, while 456 proteins were more highly expressed in *L. infantum* MP flagella. Among these, five virulence factors located on the flagella surface were identified, representing potential targets for vaccine development and drug therapy. Characteristics of the detected virulence factor proteins, including abundance, log_2_(La/Li) ratios, classification, and vaccine trial references, are summarized in Table S4. Notably, all of these surface virulence factors are encoded by multigene families [20,23,25,36,37]. Specifically, we identified 27 amastins, 17 CPs, 17 PSAs, 6 leishmanolysins, and 12 HSPs. The abundance and relative expression of each protein are shown in Figure 4 and Table S4. Amastins and leishmanolysins were predominantly expressed in *L. infantum* flagella. In contrast, PSAs were more highly expressed in *L. amazonensis* flagella.

Delta amastins, recognized as a major virulence group [38], showed differential distribution: only four showed higher expression in *L. amazonensis*, whereas twelve were more abundant in *L. infantum*. Overall, the total abundance of delta amastins in the *L. infantum* flagella was 3.9-fold greater than in *L. amazonensis*. Intriguingly, Table S4 reveals that only three of the 27 identified amastins have been evaluated as vaccine candidates [39,40,41,42,43]. Similarly, while three cysteine peptidases (CPs) enriched in *L. amazonensis* and no *L. infantum* flagella-enriched CP have been tested as vaccine antigens, the majority of the abundant CPs remain uninvestigated for vaccine development [44,45,46,47,48,49,50,51]. Furthermore, our study identified five heat shock protein (HSP) families in the MP flagellar surface proteome, predominantly HSP70 (4 members) and HSP90 (3 members). Although the total abundance of these proteins did not differ significantly between the two species, their isoform compositions showed distinct interspecific differences. Crucially, despite prior testing of one HSP20 and two HSP70 proteins, the most abundant HSPs identified here have not yet been assessed [52,53,54,55]. In line with this trend, eight parasite surface antigen (PSA) proteins have been investigated as vaccine antigens, with the exception of the most abundant isoform (PSA-34S) [56,57,58,59,60,61]. Likewise, three leishmanolysins have been tested, yet the bulk of the highly abundant leishmanolysins in our dataset remain untested [41,42,54,62,63,64,65,66]. Conclusively, our findings underscore that the majority of highly abundant virulence factors have yet to be evaluated as potential vaccine candidates.

## 4. Discussions

The flagellum is the first part of the parasite to interact with the host cell. Therefore, it may hold a key to understanding how three different forms of leishmaniasis develop, and the flagella surface protein could be a vaccine target to induce sterile immunity. However, the species-specific expression profiles of flagellar proteins across the three major leishmaniasis pathways have not yet been investigated in depth. A previous comparative genomic study reported that over 99.5% of genes were syntenic between *L. mexicana* and *L. amazonensis*, reflecting their close evolutionary relationship as members of the same species complex [67,68]. However, comparing the flagellar proteomes was insufficient to identify specific candidates potentially responsible for the divergent MCL phenotypes of the two species. Taken together, our results demonstrate that *L. infantum* has a distinct proteomic signature that clearly sets it apart from *L. mexicana* and *L. amazonensis*. These findings provide molecular insights that may help clarify species-specific biological behaviors in *Leishmania*. Notably, the enrichment of amino acid biosynthesis pathways suggests that *L. infantum* possesses distinct metabolic adaptations in its flagella-associated proteome compared to the other two species. Several key enzymes involved in amino acid biosynthesis [including cysteine synthase (LINJ_36_3750), glutamine synthetase (LINJ_06_0370), a putative threonine synthase (LINJ_14_0350), and serine hydroxymethyl transferase (SHMT-S)] were significantly more abundant in *L. infantum* than in *L. amazonensis*. These findings point to an enhanced anabolic metabolism and an increased capacity for amino acid production in *L. infantum*. Crucially, alongside carbohydrates, amino acids serve as vital energy sources for *Leishmania* parasites [69]. Being auxotrophic for multiple essential amino acids, *Leishmania* depends on the Amino Acid Permease 3 (AAP3) transporter to acquire host-derived L-arginine, which is critical for its proliferation [70]. Crucially, the parasites’ dependence on these host-derived essential nutrients can vary in a species-specific manner. Furthermore, the enrichment of purine metabolism is noteworthy, as *Leishmania* parasites are purine auxotrophs that rely entirely on host-derived purines for growth and survival [71]. Notably, several key enzymes involved in purine and nucleotide metabolism [including adenosine kinase (LINJ_30_0940), GMP synthase (LINJ_22_0013), and adenylosuccinate lyase (LINJ_04_0440)] were more highly expressed in *L. infantum*. This elevated abundance may reflect distinct capabilities in purine utilization and nucleotide biosynthesis between species [72]. Concurrently, enrichment of the pentose phosphate pathway (PPP) points to species-specific differences in NADPH production and oxidative stress defense [73]. As the PPP is a major source of NADPH in *Leishmania*, these findings suggest potential differences in the mechanisms used by *L. infantum* and *L. amazonensis* to adapt to host-induced oxidative stress. The fact that most PPP-associated proteins were upregulated in *L. infantum* further suggests an enhanced metabolic capacity to support nucleotide metabolism and cellular adaptation. Collectively, our KEGG pathway enrichment analysis indicates that the proteomic differences among these species are primarily concentrated in pathways associated with amino acid and purine metabolism, central carbon metabolism, and the pentose phosphate pathway. These metabolic distinctions suggest that *L. infantum* may employ host-adaptation strategies that differ from those of *L. mexicana* and *L. amazonensis*, thereby highlighting potential metabolic vulnerabilities that could be exploited to develop species-specific therapeutic interventions.

Virulence factors, defined as pathogen-produced molecules essential for pathogenesis but not basal viability [74], directly govern *Leishmania* infectivity and disease severity. Among these, delta-amastins are critical for survival within phagocytic macrophages; their reduced gene copy numbers in species residing in non-phagocytic cells, coupled with the impaired growth observed in knockdown mutants, underscore their essentiality [38,75]. Furthermore, amastins are recognized as promising vaccine candidates across related trypanosomatids [76]. Similarly, CPs, including the calpain-like family, mediate tissue penetration and immune modulation, making them well-established chemotherapeutic and vaccine targets for various parasitic diseases [24]. We identified seventeen CPs, predominantly calpain-like (65%), which are implicated in signal transduction and cytoskeletal remodeling [37,77]. Notably, CPB regulates leishmanolysin (GP63) [78], a matrix metalloprotease that facilitates host cell uptake and suppresses immune responses by cleaving C3b and CXCL10 [79]. Complementing GP63, PSA-2 promotes intracellular survival by inhibiting complement-mediated lysis and binding to macrophage CR3 receptors [80,81]. *Leishmania* HSPs also play pivotal roles in adaptation and persistence [19,23]. Like CPs, HSPs are extensively explored vaccine candidates across various species [82,83,84,85], and their co-administration with GP63 has been shown to enhance Th1-type protective efficacy [86]. Notably, in mammalian systems, members of the HSP70 and HSP90 families have been reported to localize to the surface of cancer cells, where they mediate interactions with immune cells [87,88]. The host immune system likely targets these functionally essential virulence factors; in response, parasites may have evolved immune evasion mechanisms by expanding gene copy numbers to amplify antigenic diversity. Remarkably, these multigene-family virulence factors constituted over 10.5% of the top 100 flagellar membrane proteins, compared to only ~5% among less abundant proteins (Table S3). This distributional bias highlights their functional significance in flagellar-host receptor interactions.

Previous comparative proteomics studies indicated that *L. infantum* exhibits more robust pre-expression of HSPs and amastins than *L. amazonensis*. While both species express CPs and leishmanolysin (GP63), *L. amazonensis* displays an earlier and more substantial abundance of GP63, whereas *L. infantum* shows elevated PSA-like protein expression during the stationary phase [15]. Furthermore, a flagellar proteomic study of *L. amazonensis* promastigotes reported differential localization of virulence factors, with GP63 and PSA concentrated in the flagellum, underscoring their roles in host interactions. In contrast, our findings reveal a distinct protein localization pattern: amastins and GP63 are preferentially enriched in the flagellar fraction of *L. infantum*, whereas PSA expression is significantly higher in that of *L. amazonensis*. Additionally, the high abundance of HSPs and CPs on the flagellar membrane suggests potential moonlighting functions at this site.

Given that quantitative flagellar proteomic analyses have not been previously reported for these species, our results identify unique flagellar protein profiles that diverge from whole-cell proteomes, in which cell-body proteins typically predominate. Notably, our species-specific expression profiling of multigene-family virulence factors associated with MP flagella provides a comprehensive dataset that may inform the future identification of potential vaccine antigens and therapeutic targets (Table S4). However, the primary contribution of this study lies not merely in identifying new antigens but in providing quantitative evidence of species-specific differences in flagellar localization to inform antigen prioritization. Historically, in the absence of comprehensive quantification, low-abundance proteins have often been prioritized as vaccine candidates. Ultimately, subsequent antibody-mediated functional assays will elucidate the role of these abundant flagellar surface factors in driving specific clinical manifestations, thereby accelerating the development of next-generation vaccines.

## Data Availability

The mass spectrometry proteomics data have been deposited to the proteomexchange consortium via the pride partner repository with the dataset identifier pxd070964 and 10.6019/pxd070964 with project name “analysis of flagella proteins in three different leishmaniasis pathways” [89]. Reviewers can access the dataset by using ‘reviewer_pxd070964@ebi.ac.uk’ as the id and ‘ycsgz6uvu7go’ as the password.

## Supplementary Materials

The following supporting information can be downloaded at: https://www.mdpi.com/article/10.3390/microorganisms14071411/s1, Table S1. Amino acid sequences of the detected peptides; Table S2. Relative abundance of Leishmania flagella proteins; Table S3. Flagellar membrane protein abundance; Table S4. Five multigene-family flagella-surface virulence factors.
