# Supplementary material for: Insights into Divergent Leishmaniasis Pathogenesis: A Quantitative Flagellar Proteomic Comparison of L. mexicana, L. amazonensis, and L. infantum
Source: Microorganisms. 2026 Jun 26;14(7):1411. doi: 10.3390/microorganisms14071411 (PMC13414411; doi:10.3390/microorganisms14071411)
Supplement: Supplementary file 1 [file microorganisms-14-01411-s001.zip › Supplementary Information_Microorganisms.pdf]

Supplementary Information

Table S1. Amino acid sequences of the detected peptides

Table S2. Relative abundance of *Leishmania* flagella proteins.

Table S3. Flagellar membrane protein abundance. Differentially expressed flagellar membrane proteins (> 2-fold change) for each species are listed in descending order of relative abundance. The 100 most abundant flagellar proteins in each species are highlighted.

Table S4. Five multigene-family flagella-surface virulence factors. All detected proteins are listed in descending order of log2(La/Li) values. Bold text highlights the two most abundant proteins in each species with a >2-fold difference. Previous vaccine trials are cited in the references.
